# Effect of aromatic oils on the expression of some virulence-associated and antimicrobial resistance genes of *Escherichia coli* isolated from broilers

**DOI:** 10.5455/javar.2022.i584

**Published:** 2022-06-26

**Authors:** Walid Hamdy Hassan, Hala Sayed Hassan Salam, Wafaa Mohamed Hassan, Salama Abohamra Sayed Shany, Ghada Sayed Ibrahim Osman

**Affiliations:** 1 Department of Bacteriology, Mycology and Immunology, Faculty of Veterinary Medicine, Beni-Suef University, Beni-Suef, Egypt; 2 Microbiology-Reference Laboratory of Quality Control on Poultry Production, Animal Health Research Institute AHRI, Giza Governorate, Egypt; 3 Department of Poultry Diseases, Faculty of Veterinary Medicine, Beni-Suef University, Beni-Suef, Egypt; 4 Department of Bacteriology, Animal Health Research Institute, Beni-Suef Laboratory, Agricultural Research Center, Beni-Suef, Egypt

**Keywords:** *Escherichia coli*, broiler, virulence, resistance, aromatic oils

## Abstract

**Objectives:**

This study aimed to prove the effects of *Escherichia coli* isolates isolated from diseased broilers to form biofilms, describe their antimicrobial sensetivity, and determine the effect of allicin and cinnamon essential oils on the expression of some genes (*fim*H, *int*1, and *lux*S) through quantitative polymerase chain reaction (q-PCR).

**Materials and Methods:**

140 samples were obtained from diseased broilers in Beni-Suef Governorate, Egypt. These samples were examined by conventional bacteriology methods to detect the causative agent. The antimicrobial susceptibility of the isolated bacteria was assessed using the disc diffusion method, The ability of yeast extract-casamino acids Congo Red Agar to generate phenotypic biofilms was next tested. The presence of resistance and virulence genes in some multidrug resistant isolates was genotypically investigated. The antibacterial effects of allicin and cinnamon oil were evaluated against the growth of multidrug-resistant *E. coli*. Finally, q-PCR was utilized to assess changes in some genes’ expression.

**Results:**

*Escherichia coli* was isolated from 61 samples (43.6%). An antimicrobial susceptibility test revealed that multidrug-resistance (MDR) (could resist more than three antimicrobial classes) *E. coli* prevalence was 100%. 40.8% of isolates phenotypically produce biofilms. The detection of resistance and virulence genes by PCR showed that all tested isolates carry *aad*B, *fim*H, *int*1, *qnr*S, and *lux*S genes, while only 40% harbor *iss* genes. q-PCR showed that after treatment with allicin and cinnamon oils, gene expression went down.

**Conclusion:**

This investigation highlights that *E. coli* showed resistance against most of the tested antimicrobials; all isolates were MDR. The study showed wide dissemination of virulence and resistance genes among *E. coli*. Allicin and cinnamon oils have antimicrobial activities and could be used as alternatives to synthetic antimicrobial agents.

## Introduction

*Escherichia coli *belongs to the Enterobacteriaceae family [1]. Even though *E. coli* is a normal occupant of poultry’s digestive tract, and pathogenic serotypes have previously been reported from the intestinal tract of healthy poultry, supporting the claim that *E. coli* is frequently a secondary or opportunistic infection [2]. *Escherichia coli* can cause colibacillosis outbreaks associated with various disease conditions [3]. Stress may play a role in transmitting avian pathogenic *E. coli* from the bird’s gut to the bloodstream. It infects several internal organs and produces pericarditis, perihepatitis, salpingitis, peritonitis, and other extra-intestinal symptoms [4]. Colibacillosis can affect chickens of different ages but is more common and severely affects young birds [2]. Colisepticemia is the most prevalent form of avian colibacillosis, which causes hefty financial losses worldwide [5]. Misusage of antimicrobials and their prophylactic application in the poultry field are considered to be the causes of drug resistance [6]. Increased antimicrobial resistance patterns pose an important public health concern [7]. 

Therefore, the drug of choice should be chosen based on its sensitivity, which may be determined by lab examination and employed for an accurate dose and course. Other than resistance gene expression, bacteria can protect themselves by producing vast amounts of extracellular polymeric substance (EPS) during the biofilm formation process [8]. This EPS plays a critical role in antibiotic resistance and host immunity [9]. Quorum sensing (QS), a type of cell-to-cell communication in which an accumulation of signaling molecules in the extracellular environment causes gene expression to be controlled, initiates the biofilm-building process [10]. Several spices and plants are now prized for their antibacterial properties [11]. Consequently, there is an increased interest in using natural, non-toxic, and effective compounds as an alternative to synthetic antimicrobials. These are primarily herb and spice extracts and essential oils (EOs) [12]. The Eos antimicrobial activity might be due to the high hydrophobicity that enables them to cross the microbial cell membranes, leading to loss of function or damage of the cellular proteins, lipids, and organelles, resulting in bacterial cell death [13]. The cinnamon EO’s antibacterial activity may be attributed to its richness in cinnamaldehyde, which exhibits antibacterial properties against many pathogenic bacteria [14]. Garlic contains diallyl sulfide, which penetrates and dissolves the bacteria’s protective bio-layer, eventually killing them [15]. Allicin, a component of garlic, contains a variety of thiosulfinates responsible for its antibacterial properties [16]. The goal of this study was to find out if *E. coli* from sick broilers in Beni-Suef Governorate, Egypt, had the ability to form biofilms and if they were sensitive to antimicrobials. They also wanted to use quantitative polymerase chain reaction (q-PCR) to find out how allicin and cinnamon essential oils affect the expression of certain genes (*fim*H, *int*1, and *lux*S).

## Materials and Methods

### Samples

One hundred forty samples were taken aseptically from broiler chicks aged one to 35 days and collected from Beni-Suef governorate, Egypt, between January 2020 and April 2020. These chicks suffered from respiratory manifestations and/or gastrointestinal problems. Swabs from the heart, lung, liver, and yolk sac that showed gross lesions were used for bacteriological isolation.

### Bacteriological examination

**Bacteriological and biochemical identification of isolated *E. coli***

The samples taken were inoculated under aseptic conditions into buffered peptone water and incubated aerobically at 37°C for 24 h. A loopful from the media was streaked onto MacConkey agar medium. Then all plates were incubated aerobically at 37°C for 24 h. Suspicious colonies for *E. coli* appear on MacConkey agar as pinkish colonies. One colony representing a single typical colonial appearance and morphological character was picked up for inoculation onto eosin methylene blue agar medium. Bacterial smears were stained by Gram’s staining technique. Biochemical identification was carried out according to Quinn et al. [17], including oxidase test, indole production, methyl red test, Voges Proskauer test, citrate utilization test, sugar fermentation test, and hydrogen sulfide (H_2_S) production on TSI medium, and urea hydrolysis test.

**Antimicrobial sensitivity testing of *E. coli* isolates**

The antibiogram was done by using the disk diffusion method. Antimicrobial discs included amoxicillin/clavulanic acid (30 μg), cefotaxime (30 μg), doxycycline (30 μg), fosfomycin (200 μg), gentamicin (10 μg), nitrofurantoin (300 μg), norfloxacin (10 μg), oxytetracycline (30 μg) and sulphamethoxazole/trimethoprim (25 μg), represented different antimicrobial classes of veterinary concern. The recorded zones were interpreted according to CLSI [18]. Multidrug-resistance (MDR) was defined as resistance to three or more antimicrobial classes according to Magiorakos et al. [19].

**Biofilm formation of *E. coli* isolates**

The test was done according to Zhou et al. [20]. YESCA/ CR agar plates were inoculated and incubated aerobically for 48 h at 26°C. Positive results were indicated by dark red, red, or light red colonies, while negative results were pink, light pink, or white colonies.

### Detection virulence-associated and resistance genes in E. coli

Five MDR *E. coli* isolates were subjected to PCR to identify three resistance-associated genes (*aad*B, *int*1 and *qnr*S) and three virulence-related genes (*fim*H,* iss,* and* lux*S). The QIAamp DNA mini kit was used to extract DNA, as stated by the manufacturer’s instructions and using their specific forward and reverse primers as indicated in Table 1. The DNA of the positive control was taken from an* E. coli *field isolate that was proven to be positive in RLQP (Reference Laboratory for Veterinary Quality Control on Poultry Production, Dokki, Giza, Egypt). A negative control, on the other hand, is a PCR mixture without the DNA template.

### Evaluation of the antibacterial effects of allicin and cinnamon oils on the growth of multidrug-resistant E. coli isolates

Allicin and cinnamon oils were investigated for their antimicrobial activities against 15 MDR *E. coli *isolates [21]. Essential oils were prepared as 10% (10,000 ppm) in DMSO as the original solution. Allicin oil (diallyl disulfide, 99.9%) was obtained from Seven Seas Pure Chemical Industries, Ltd., China. It was prepared at 500, 250, and 125 ppm concentrations in tryptone soya agar. Cinnamon oil (Aldrick, Sigma) was prepared at 125, 63, 32, 16, and 8 ppm concentrations in tryptone soya agar.

### Qualitative real-time PCR for E. coli isolates

Three MDR *E. coli* strains were subjected to quantitative RT-PCR before and after treatment with 125 ppm allicin and 32 ppm cinnamon to estimate variations of *fimH *luxS, and *Int*1 genes. A quantitative RT-PCR was prepared using the Quantitect SYBR Green PCR kit. Primer sequences and probes used in SYBR Green real-time PCR are illustrated in Table 2.

## Results

### Prevalence of E. coli

Sixty-one *E. coli* isolates were isolated from 140 samples collected from diseased broilers with an incidence of 43.6 %.

### Antimicrobial sensitivity testing of recovered E. coli 

The explored *E. coli *isolates (*n* = 61) showed high sensitivity rates to fosfomycin (85.2%) and nitrofurantoin (80.3%). On the contrary, high resistance rates were recorded for amoxicillin-clavulanic acid (100%), cefotaxime (98.4%), oxytetracycline (90.2%), sulfatrimethoprime (90.2%), and gentamicin (88.5%). The detailed results of antimicrobials are included in Table 3. Moreover, all the investigated *E. coli* isolates (100%) were MDR.

**Table 1.** Primers of virulence-associated and resistance genes used in PCR.

Target gene	Primers direction	Primers sequences (5′-3′)	Amplified product size (bp)	Reference
*Aad*B	F	GAG CGA AAT CTG CCG CTC TGG	319	[61]
	R	CTG TTA CAA CGG ACT GGC CGC		
*Fim*H	F	TGC AGA ACG GAT AAG CCG TGG	508	[62]
	R	GCA GTC ACC TGC CCT CCG GTA		
*Iss*	F	ATG TTA TTT TCT GCC GCT CTG	266	[63]
	R	CTA TTG TGA GCA ATA TAC CC		
*Int*1	F	CCT CCC GCA CGA TGA TC	280	[35]
	R	TCC ACG CAT CGT CAG GC		
*Lux*S	F	ATG CCG TTG TTA GAT AGC TTC A	513	[64]
	R	GAT GTG CAG TTC CTG CAA CTT C		
*Qnr*S	F	ACG ACA TTC GTC AAC TGC AA	417	[65]
	R	TAA ATT GGC ACC CTG TAG GC		

**Table 2.** Oligonucleotide primers and probes used in SYBR Green real time PCR.

Gene	Primers direction	Primer sequence (5‘-3‘)	Reference
*E. coli 16S* *rRNA *	F	GCT GAC GAG TGG CGG ACG GG	[66]
	R	TAG GAG TCT GGA CCG TGT CT	
*Fim*H	F	TGC AGA ACG GAT AAG CCG TGG	[62]
	R	GCA GTC ACC TGC CCT CCG GTA	
*Lux*S	F	ATG CCG TTG TTA GAT AGC TTC A	[64]
	R	GAT GTG CAG TTC CTG CAA CTT C	
*Int*1	F	CCT CCC GCA CGA TGA TC	[35]
	R	TCC ACG CAT CGT CAG GC	

### Biofilm formation of E. coli on YESCA/CR agar

Out of 61*E. coli* isolates, 37 (60.7%) were positive on YESCA/ CR agar, while 24 were negative on YESCA/CR agar.

### PCR of E. coli

**Figure 1.**
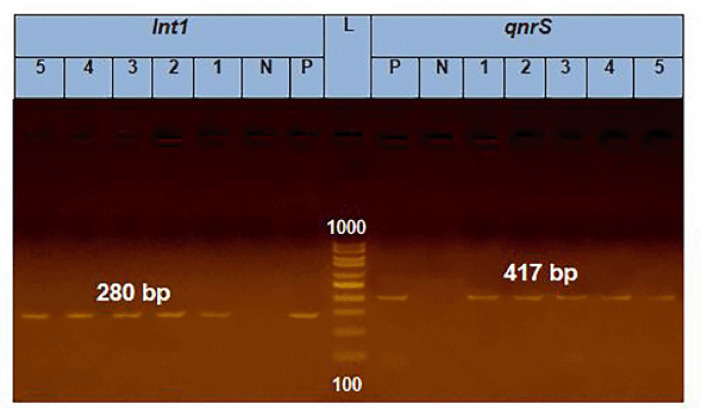
PCR amplification of the *int*1 gene at 280bp and the* qnr*S gene at 417bp fragments. Lanes 1–5 showed positive amplification of int1 and *qnr*S genes. P = Positive control; N = negative control; L = 100-bp DNA molecular size ladder.

**Figure 2.**
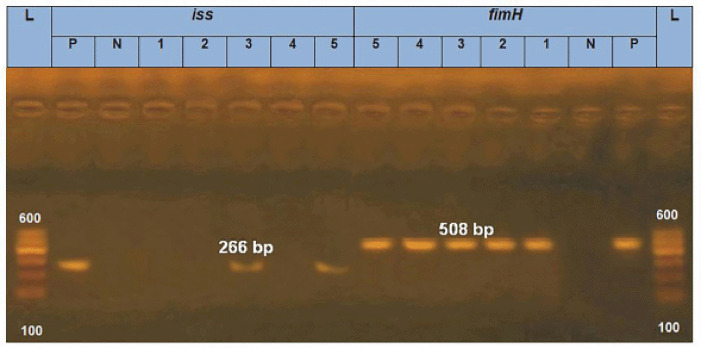
PCR amplification of the *iss* gene at 266 bp and the *fim*H gene at 508 bp fragments. Lanes 1–5 showed positive amplification of* fim*H gene, and Lanes 3, 5 showed positive amplification of *iss *gene. P = Positive control; N = negative control; L = 100-bp DNA molecular size ladder.

**Figure 3.**
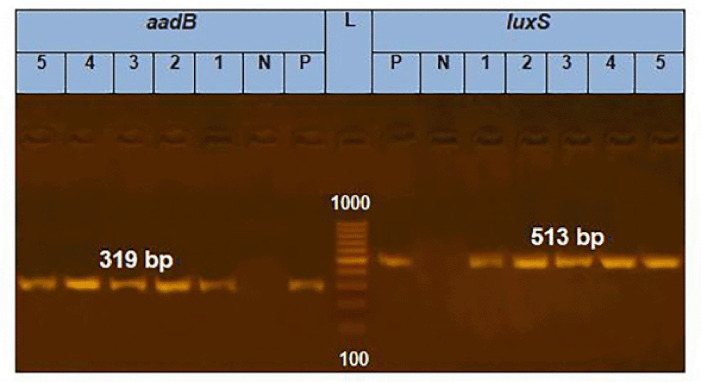
PCR amplification of the *aad*B gene at 319 bp and the *lux*S gene at 513 bp fragments. Lanes 1–5 showed positive amplification of *aad*B and* lux*S genes. P = Positive control; N = negative control; L = 100-bp DNA molecular size ladder.

All of the tested *E. coli* isolates (*n* = 5) carried *aad*B, *int*1 and *qnr*S genes (100%) in terms of the resistance genes. In relation to the virulence-associated genes, all the tested* E. coli* isolates (100%) had *fim*H, and* lux*S; meanwhile, two tested *E. coli* isolates (40%) harbored *iss* gene. (Figs. 1–3).

### Antibacterial effects of allicin and cinnamon oils on multidrug-resistant E. coli isolates

Allicin oil fully inhibited the growth of all *E. coli* isolates (*n* = 15) (100%) at concentrations of 250 and 500 ppm. On the contrary, 125 and 63 ppm of allicin concentrations lead to retarded and little growth of *E. coli *(Table 4).

Cinnamon oil fully inhibited the growth of all *E. coli* isolates (*n* = 15) (100%) at concentrations of 125 ppm and 63 ppm, while at 32 ppm it inhibited 4 isolates only (26.7%) of *E. coli*. On the other hand, the 16 and 8 ppm concentrations did not kill the *E. coli* isolates, but they grew slowly and not at all (Table 5).

**Table 3.** Antimicrobial susceptibility of *E. coli* isolated from broiler chicks (*n* = 61).

Antimicrobial class	Antimicrobial disk	Susceptible		Intermediate		Resistant	
		No.	%	No.	%	No.	%
β-lactamase stable	Amoxicillin-clavulanic acid	0	0	0	0	61	100
Cephalosporins	Cefotaxime	0	0	1	1.6	60	98.4
Tetracyclines	Doxycycline	7	11.5	7	11.5	47	77
	Oxytetracycline	2	3.3	4	6.5	55	90.2
Fosfomycins	Fosfomycin	52	85.2	0	0	9	14.8
Aminoglycosides	Gentamicin	3	4.9	4	6.6	54	88.5
Nitrofurans	Nitrofurantoin	49	80.3	10	16.4	2	3.3
Quinolones	Norofloxacin	3	4.91	11	18.03	47	77.04
Potentiated sulfonamide	Sulphamethoxazole/trimethoprim	3	4.9	3	4.9	55	90.2

### Quantitative real time-PCR (RT-PCR) for multidrug-resistant E. coli before and after treatment with allicin and cinnamon oils

**Figure 4.**
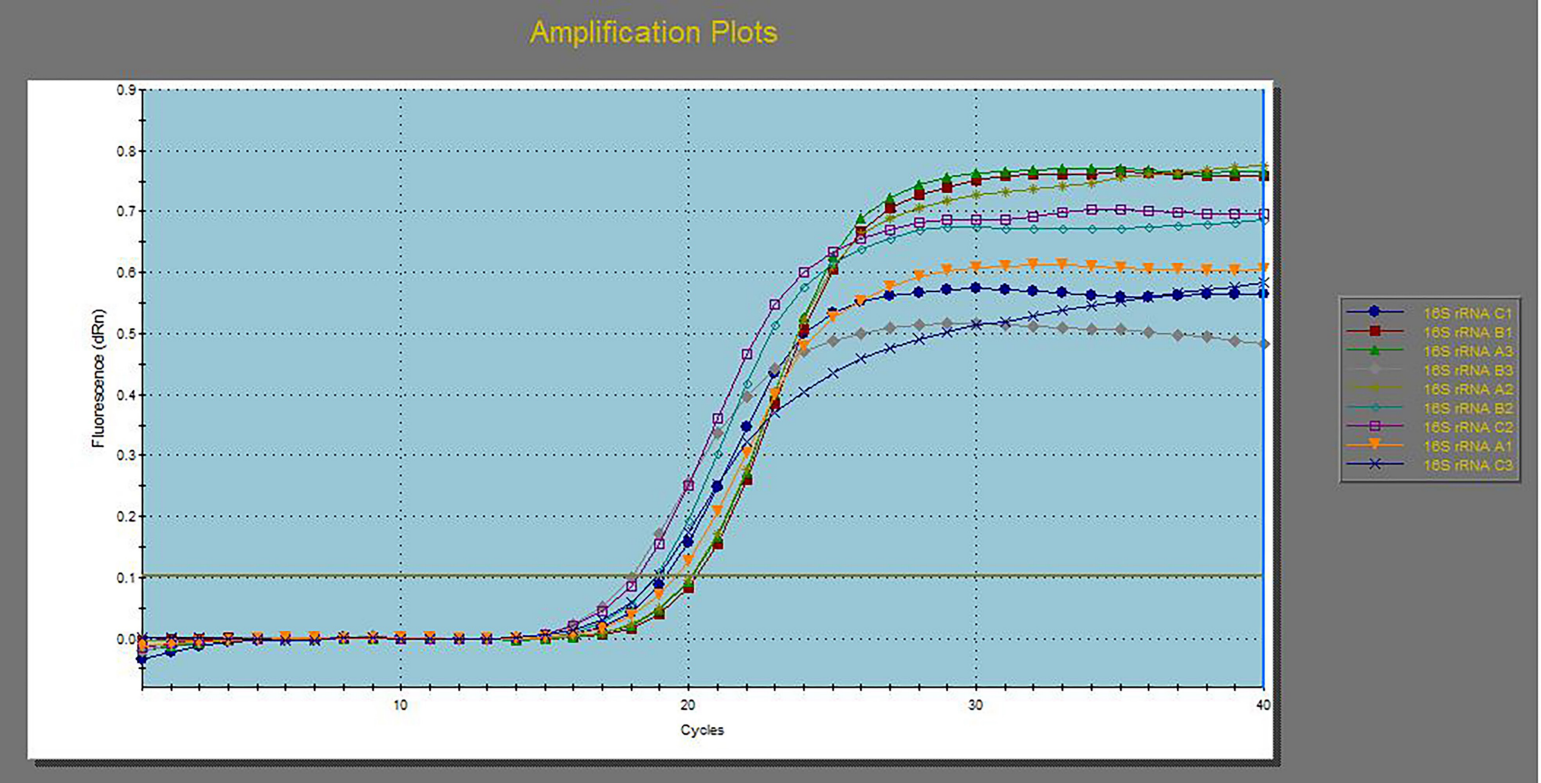
Down-regulation in *16s rRNA* gene from 3 MDR *E. coli* before and after exposure to 125 ppm allicin and 32 ppm cinnamon oils.

**Figure 5.**
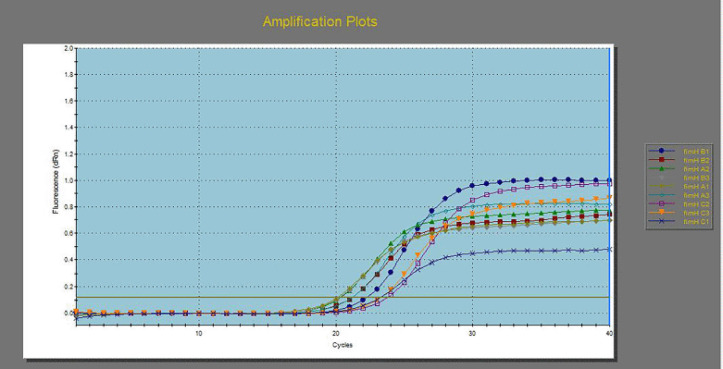
Down-regulation in *fim*H gene from 3 MDR *E. coli* before and after exposure to 125 ppm allicin and 32 ppm cinnamon oils.

**Figure 6.**
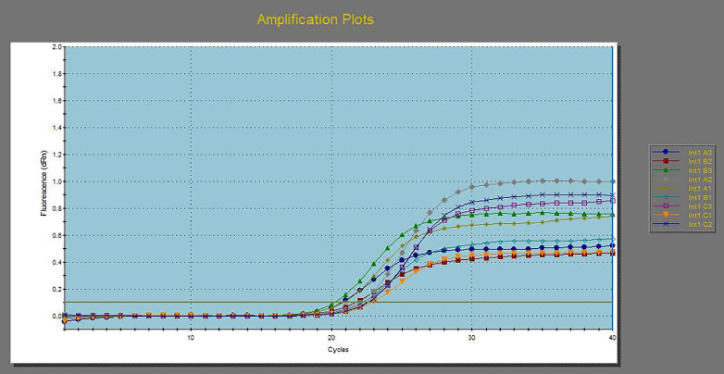
Down regulation in *int*1 gene from 3 MDR *E. coli* before and after exposure to 125 ppm allicin and 32 ppm cinnamon oils.

**Figure 7.**
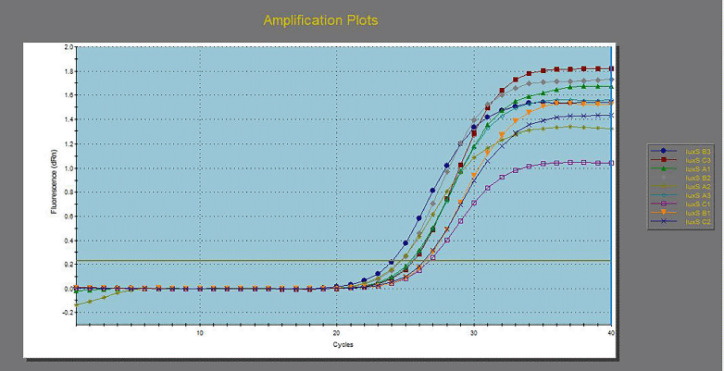
Down regulation in *lux*S gene from 3 MDR *E. coli* before and after exposure to 125 ppm allicin and 32 ppm cinnamon oils.

Table 6 and Figures 4–7 illustrated the down-regulation in fold change of *E. coli* for the *fim*H gene after exposure to 125 ppm allicin and 32 ppm cinnamon oil were 0.4061 and 0.1397, respectively, for the first *E. coli *isolate; 0.4118 and 0.0693 for the second *E. coli* isolate; and 0.3763 and 0.1768, respectively, for the third *E. coli* isolate. In the same context, down regulations in fold change for* Int*1gene were 0.6974 and 0.3099 for the first *E. coli *isolate, 0.6417 and 0.2755, respectively, for the second *E. coli* isolate, and 0.5105 and 0.2755 for the third *E. coli* isolate. On the other hand, down regulations in fold change for *lux*S were 0.7120 and 0.3121, respectively, for the first *E. coli *isolate; 0.6156 and 0.1934, respectively, for the second *E. coli* isolate; and 0.8293 and 0.3368, respectively, for the third *E. coli* isolate.

**Table 4.** The antibacterial effect of allicin oil on *E. coli* isolates (*n* = 15).

Bacterial isolates	Allicin oil concentration							
	500 ppm		250 ppm		125 ppm		63 ppm	
	No.	%	No.	%	No.	%	No.	%
* E. coli*	0	0	0	0	15	100	15	100

No.: Number of the affected (growth inhibited) isolates.

%: was calculated according to the number (No.) of tested bacterial isolates.

**Table 5.** The antibacterial effect of cinnamon oil on *E. coli* isolates (*n* = 15).

Bacterial isolates	Cinnamon oil concentration									
	8 ppm		16 ppm		32 ppm.		63 ppm		125 ppm	
	No.	%	No.	%	No.	%	No.	%	No.	%
*E. coli *	0	0	0	0	4	26.7	15	100	15	100

No.: Number of the affected (growth inhibited) isolates.

%: was calculated according to the number (No.) of tested bacterial isolates.

**Table 6.** RT-PCR analysis for *E. coli* isolates before and after treatment Allicin and cinnamon oils.

*E. coli* Sample No.	Treatment	Sample ID	*16S rRNA*	*fimH*		*luxS*		*Int1*	
			CT	CT	Fold change	CT	Fold change	CT	Fold change
1	Control	A1	19.87	20.38	-	23.06	-	21.46	-
	Allicin	B1	20.52	22.33	0.4061	24.20	0.7120	22.63	0.6974
	Cinnamon	C1	19.86	23.21	0.1397	24.73	0.3121	23.14	0.3099
2	Control	A2	20.11	20.92	-	22.95	-	22.18	-
	Allicin	B2	19.44	21.53	0.4118	22.98	0.6156	22.15	0.6417
	Cinnamon	C2	18.91	23.57	0.0693	24.12	0.1934	22.84	0.2755
3	Control	A3	20.34	21.60	-	23.10	-	21.59	-
	Allicin	B3	18.10	20.77	0.3763	21.13	0.8293	20.32	0.5105
	Cinnamon	C3	19.62	23.38	0.1768	23.95	0.3368	22.73	0.2755

## Discussion

*Escherichia coli* infection causes severe economic losses in poultry production [22]. In this study, *E. coli* prevalence was studied in diseased broilers. Sixty-one isolates were recovered from the studied cases, with 43.6%. This finding was nearly identical to that reported by Awad et al. [23], who revealed only 28 *E. coli* isolates out of 54 (51.9%) in Egypt’s northern delta during their first two weeks of life.

The disc diffusion method was used to study the *in vitro* susceptibility patterns of *E. coli* isolates against nine different antimicrobial drugs from eight other classes. The most effective antimicrobials on *E. coli *were fosfomycin (85.2%) and nitrofurantoin (80.3%). The antimicrobial amoxicillin-clavulanic acid showed the highest resistance (100%), followed by cefotaxime (98.4%), oxytetracycline, sulfatrimethoprime (90.2% for each), and gentamicin (88.5%).

The nearly same result of high resistance recorded by Amer et al. [24] oxytetracycline resistance was 85%. The reported high rates of resistance could be due to the use of antimicrobial drugs in a frequent manner [25]. *Escherichia coli *frequently carries many drug resistance plasmids and can easily transfer those plasmids to other species when stressed*.* *E. coli* can receive and transfer plasmids from one bacteria to another when there is a mixing of species in the intestines [26]. In this study, 100% of *E. coli* isolates were MDR. These findings corroborate those obtained by many authors in Egypt who found that *E. coli* isolates were 100% MDR [7,24].

Biofilm formation is one of bacteria’s resistance and virulence strategies [27], in which biofilms are surface-attached microbial cells encapsulated in an extracellular matrix that are much more resistant to antimicrobial treatments than non-adherent planktonic cells. Biofilm formation interferes with antimicrobial susceptibility. Bacteria become resistant via a variety of mechanisms, including antimicrobial interactions with biofilm matrix components, slower growth rates, and the various actions of specific antibiotic genetic determinants, which can increase antimicrobial resistance by up to 1,000 fold [28]. low doses of certain antibiotics can induce biofilm formation [27]. Reichhardt et al. [29] stated that Congo red on YESCA agar binds to curliated whole cells without bacterial growth inhibition and can be used to measure whole-cell curliation in which *E. coli* accumulates extra-cellular sticky amyloid fibers known as curli, which enables bacterial adherence and aids biofilm development.

In the present study, 60.7% of *E. coli* isolates were phenotypically positive biofilm formers on YESCA/CR agar. These results are higher than those of Amer et al. [24], 35.7%, and Raheel et al. [30], 46.6%, and lower than that obtained by Wang et al. [31], 81.64%.

A gentamicin resistance encoding gene, *aad*B*,* was identified in all tested* E. coli*. The high prevalence of the *aad*B gene in selected* E. coli *isolates was nearly similar to Zając et al. [32], which reported *aad*B in 93.8% of tested *E. coli *isolates. But lower percentages were obtained by Kim et al. [33] and Nhung et al. [34]: 2.5% and 12.3%, respectively, while Radwan et al. [12] did not detect the *aad*B gene in any isolates.

The antimicrobial resistance in APEC could be due to class 1 integron gene cassettes [33]. There is a strong relationship between the incidence of integrons and the phenotypes of multiresistance. Integrons carrying antimicrobial resistance genes have recently been more common in *E. coli* isolates and act as a vehicle for antimicrobial resistance acquisition and spread in the environment [35]. *The qnr*S gene is a plasmid-borne quinolone resistance gene. The *qnr*S gene of quinolones in *E. coli* isolates in this study poses a threat to public health. This is because qnr-plasmids are frequently coupled with integrons and contain a variety of resistance determinants, providing resistance to a variety of antimicrobial classes, including -lactams and aminoglycosides [36]. All tested* E. coli *isolates (100%) were positive for the *int* and *qnr*S genes. The high prevalence of the *int*1 gene agrees with Ibrahim et al. [37] and is lower than Tran et al. [38], at 22.2%. The results of *qnr*S in the current study are lower than the result reported as 31.8% by Szmolka et al. [39].

*The fim*H gene is one of the adhesion encoding genes. Fim H is an APEC virulent factor that encodes a fimbrial adhesion (type 1 pili) and promotes bacterial colonization, invasion, and biofilm formation [40]. The *fim*H gene was found in all tested isolates in the present study. The fimH results agreed with Wu et al. [41], who discovered that 100% of the tested isolates’ genes were fimH positive.

*Iss *is a serum survival gene. This gene is significantly associated with APEC strains, and it is discovered in APEC significantly more frequently than commensal *E. coli* [42]. The presence of the *iss* gene in conjugative Col V plasmids suggests a link to APEC pathogenicity [43]. A similar result was reported by Moon et al. [44], who recorded *iss* as 41%.

In response to various environmental stimuli, *E. coli* uses QS to respond to their cell population and coordinate virulence gene expression [45]. Bacteria communicate via specialized chemical molecules to control the production of virulence factors and antimicrobial-resistant determinants to regulate their action against the host [46]. *lux*S (luciferase genes) has been found to have an essential function in regulating bacterial behavior [47]. It is also crucial for biofilm development, adhesion, and gene expression [31,48]. The LuxS/AI-2 QS system implicates the synthesis of cell signaling molecules via *luxS*-based autoinducer-2 (AI-2) [49]. The current work found the *lux*S gene in 100% of the tested isolates.

As a result of their anti-QS activity, some of these EOs demonstrate an inhibitory effect against resistant bacterial isolates, which could help lower the *in vivo* virulence and pathogenicity of drug-resistant bacteria [50]. The mode of action of the EOs depends on the cell wall degradation, cell membrane damage, cytoplasm coagulation, damaging the proteins of the membrane and increasing permeability, leading to cell content leakage (ions and other cell contents), decreasing the motive force of protons, decreasing the intracellular ATP pool due to decreased ATP synthesis and enhanced hydrolysis that is separate from the increased membrane permeability, and decreasing the membrane potential due to increased membrane permeability [50].

In this study, allicin showed complete growth inhibition of all the tested *E. coli* isolates (100%) at concentrations of 250 and 500 ppm. Allicin works primarily by blocking enzymes necessary for bacterial metabolism and interferes with RNA synthesis [51]. Allicin reacts with enzyme-containing thiol, thus inhibiting the Acetyl Co-A forming system and disturbing DNA synthesis and protein synthesis [52]. The present findings agree with Babu et al. [53], who reported that the MIC of *E. coli* was 1:1,000 but lower than the results obtained by Banerjee and Sarkar [54], in which the MIC was 10 mg ml^−1^ for *E. coli.*

Cinnamon showed complete growth inhibition of all the tested *E. coli* isolates (100%) at concentrations of 125, and 63 ppm. These findings were strengthened by those described by Lu et al. [55], who investigated the antibacterial action of cinnamon oil on *E. coli* isolates’ viability and verified that their MIC ranged from 100 to 400 ppm; nonetheless, the present result was lower than that obtained by Duan and Zhao [56], in which the MIC of cinnamon against *E. coli* was 1000 ppm.

The antibacterial property of cinnamon EO may be attributed to its richness in cinnamaldehyde (the foremost constituent of cinnamon EO, comprising 85%). This aromatic aldehyde exhibits antibacterial properties against a wide variety of pathogenic microorganisms [14]. Cinnamaldehyde is a great electro-negative compound that can interrupt the biological mechanisms, including electron transport due to containing the carbonyl group that can bind to metal ions, sulfhydryl groups, and nitrogen-containing components such as proteins and nucleic acids, inhibiting the action of amino acid decarboxylases and consequently inhibiting the microorganisms’ growth [57]. Cinnamon oil contains benzoic acid, benzaldehyde, and cinnamic acid, which have a lipophilic property that is responsible for its antibacterial activity as they interfere with the lipid bilayer of the cytoplasmic membrane of the bacteria, causing it to lose its integrity [58]. This backs up the idea that EOs are effective antimicrobials against a wide range of pathogenic bacteria. Finally, differences in the antibacterial findings of EO and their MICs reported in different papers could be attributable to different methodologies used to determine the MIC. Also, the purity and stability of the oil may have something to do with the different results [59].

The quantitative RT-PCR results for the *E. coli fim*H virulence gene revealed that the fold changes after treatment with 125 ppm allicin and 32 ppm cinnamon were 0.4061 and 0.1397, respectively, which indicated that the gene expression for *fim*H gene after treatment had decreased to 0.41 and 0.14 folds, respectively. In the same context, the fold changes after treatment of the second *E. coli* isolate with 125 ppm allicin and 32 ppm cinnamon were 0.4118 and 0.0693, respectively, and the fold changes after treatment of the third *E. coli* isolate with 125 ppm allicin and 32 ppm cinnamon were 0.3763 and 0.1768, respectively.

The quantitative RT-PCR results for the *E. coli *int1 resistance gene showed that the fold changes after treatment with 125 ppm allicin and 32 ppm cinnamon were 0.6974 and 0.3099, respectively, which indicated that the gene expression for the *Int*1 gene after treatment had decreased to 0.70 and 0.31 folds, respectively. In the same context, the fold changes after-treatment of the second *E. coli* isolate with 125 ppm allicin and 32 ppm cinnamon were 0.6417 and 0.2755, respectively, and the fold changes after treatment of the third *E. coli* isolate with 125 ppm allicin and 32 ppm cinnamon were 0.5105 and 0.2755, respectively.

The quantitative RT-PCR results for the *E. coli lux*S virulence gene showed that the fold changes after treatment with 125 ppm allicin and 32 ppm cinnamon were 0.7120 and 0.3121, respectively, which indicated that the gene expression for the *lux*S gene after treatment had decreased to 0.71 and 0.31 folds, in that order. In the same context, the fold changes after treatment of the second *E. coli* isolate with 125 ppm allicin and 32 ppm cinnamon were 0.6156 and 0.1934, respectively, and the fold changes after treatment of the third *E. coli* isolate with 125 ppm allicin and 32 ppm cinnamon were 0.8293 and 0.3368, respectively.

Allicin and cinnamon are forms of plant-derived antimicrobial agents (PDAs) that can reduce *E. coli *virulence gene expression [60]. All of the results show that allicin and cinnamon oil could be used to make *E. coli* less dangerous [60].

## Conclusion

This investigation highlights the high resistance of *E. coli* against most of the tested antimicrobials; all isolates were MDR. The presence of both virulence and resistance genes among *E. coli*. Allicin and cinnamon oils have great antimicrobial activities and could be used as alternatives to synthetic antimicrobial agents.

## List of abbreviations

μg, microgram; *Aad*B, aminoglycoside adenylyltransferase B; Acetyl co-A, acetyle choline A; AI-2, autoinducer-2; APEC, avian pathogenic *E. coli*; ATP, adenosine triphosphate; CLSI, Clinical and Laboratory Standards Institute; Col V, colicin V; CRA, Congo Red Agar; DNA, deoxyribonucleic acid; Eos, essential oils; EPS, extracellular polymeric substance; *Fim*H, fimbrial H; h, Hour; *int*1, class 1integrons; *Iss*, increased serum survival; *lux*S, luciferase S; MDR, Multidrug resistance; MIC, minimum inhibitory concentration; PCR, Polymerase chain reaction; PDAs, Plant-derived antimicrobial agents; ppm, part per million; *qnr*S, quinolones resistance S; q-PCR, qualitative Polymerase chain reaction; QS, Quorum sensing; RLQP, Reference laboratory for veterinary quality control on poultry production; RT-PCR, real-time Polymerase chain reaction; SYBR, synergy brands; TSI, triple sugar iron agar; YESCA/ CR, Yuba Environmental Science Charter Academy|/ congo red.
